# Characteristics of proximal early gastric cancer differentiating distal early gastric cancer

**DOI:** 10.1371/journal.pone.0223284

**Published:** 2019-09-27

**Authors:** Jin Sung Koh, Moon Kyung Joo, Jong-Jae Park, Beom Jae Lee, Hoon Jai Chun, Sang Woo Lee, You-Jin Jang, Young-Jae Mok

**Affiliations:** 1 Division of Gastroenterology, Department of Internal Medicine, Korea University Guro Hospital, Korea University College of Medicine, 148, Gurodong-ro, Guro-gu, Seoul, Republic of Korea; 2 Division of Gastroenterology, Department of Internal Medicine, Korea University Anam Hospital, Korea University College of Medicine, 73, Inchon-ro, Seongbuk-gu, Seoul, Republic of Korea; 3 Division of Gastroenterology, Department of Internal Medicine, Korea University Ansan Hospital, Korea University College of Medicine, 123, Jeokgeum-ro, Danwon-gu, Ansan-si, Gyeonggi-do, Republic of Korea; 4 Department of Surgery, Korea University Guro Hospital, Korea University College of Medicine, Gurodong-ro, Guro-gu, Seoul, Republic of Korea; National Cancer Center, JAPAN

## Abstract

Previous studies reported substantial differences between proximal and distal gastric cancer, however, most of the cases included in these studies were advanced gastric cancers (AGCs). The aim of this study was to investigate the unique characteristics of proximal early gastric cancer (EGC) by comparing with distal EGC. From March 2007 to March 2016, proximal and distal EGC patients who underwent endoscopic or surgical resection at our institution were matched 1:3 according to age and sex. We retrospectively analyzed the clinical and histopathological information. A total of 368 patients were enrolled including 92 (25%) in the proximal and 276 (75%) in the distal group. The proportion of patients who underwent surgery (56.5 vs. 20.3%, *p*<0.001), undifferentiated type (38.0 vs. 19.6%, *p*<0.001), tumor size (29.5 ±19.4 vs. 20.3 ±16.8 mm, *p*<0.001) and submucosal (SM) invasion (60.9 vs. 25.7%, *p*<0.001) were significantly higher in the proximal group than in the distal group. In multivariate analysis, the proximal location of EGC was a significant risk factor for SM invasion in the total population (odds ratio [OR], 3.541; 95% confidence interval [CI], 2.053–6.110; *p*<0.001), and in subgroup with EGC < 30mm (n = 279) (OR, 5.940; 95% CI, 2.974–11.862; *p*<0.001). In conclusion, careful therapeutic decision of proximal EGC is essential due to the different histopathological characteristics such as large tumor size and higher potential for SM invasion.

## Introduction

Gastric cancer is one of the most common cancers and the third leading cause of cancer-related death worldwide [1]. If gastric cancer is diagnosed at an advanced stage or leads to distal metastasis, the chances of cure are reduced dramatically [2]. Thus, detection at an early stage followed by fast and non-invasive resection is the most important factor contributing to complete remission of gastric cancer. Over the last decade, endoscopic submucosal dissection (ESD) has been established as a standard therapeutic option for the treatment of early gastric cancer (EGC) without lymph node metastasis and lymphatic or vascular invasion [3]. Differentiated mucosal cancer without ulcerative findings and tumor size ≤20 mm are an absolute indication for ESD [4]. However, fewer than expected short-term outcomes of ESD including *en bloc* and curative resection for undifferentiated mucosal cancer and differentiated SM invasion cancer have been reported [5, 6]. Histopathological evaluation of resected specimen may reveal that EGC with minor SM invasion ≤500 μm may accompany lymphovascular invasion [7]. Thus, it is an important issue to select minute SM invasive EGC before procedure and carefully consider the options of ESD or surgery.

However, few diagnostic tools are available for the effective detection of SM invasive cancer. Pre-procedural endoscopic ultrasonography (EUS) may be used to investigate SM invasion. However, EUS is a tedious procedure, with poor image and inter-observer variation for the staging of EGC, compared with conventional white-light endoscopy (WLE) [8]. A well-trained endoscopist may predict submucosal invasion based on several suggestive findings of WLE such as irregular surface, marginal elevation and clubbing, fusion or cutting of converging folds, despite disappointing diagnostic accuracy (72–81%) [9–11]. Combining the findings of WLE and EUS or other associated factors may strengthen the predictive value of of EGC for the depth of invasion. Proximal gastric cancer exhibits different biological or clinicopathological behavior compared with distal gastric cancer [12]. However, data about the unique characteristics of proximal EGC are very limited. Therefore, we investigated the unique characteristics of proximal EGC compared with the antral EGC by focusing on the depth of invasion and other clinicopathological parameters.

## Methods

### Study population

From March 2007 to March 2016, patients who were diagnosed with EGC and underwent surgical or endoscopic resection at our institution were subdivided according to the location. Proximal EGC was defined by tumor location in the upper third of stomach and distal EGC by the lower third of stomach, according to the Japanese classification of gastric carcinoma 3^rd^ edition [13]. We excluded EGC involving esophagogastric junction (EGJ) to minimize the component of esophageal adenocarcinoma (EAC). Cases corresponding to proximal EGCs were collected. We calculated propensity score by logistic regression with respect to age and sex, and matched proximal and distal cases as 1: 3 ratio manually. We retrospectively analyzed clinical information including age, gender, family history, comorbidities, and *Helicobacter pylori* (*H*. *pylori*) infection. This study was approved by the ethics committee of Korea University Guro Hospital review board (IRB no. K2018-0919-001), and this study protocol conforms to the ethical guidelines of the 1975 Declaration of Helsinki as reflected in a prior approval by the Korea University Guro Hospital's human research committee. Written, informed consent was obtained from each patient included in the study.

### Histopathology

ESD and surgery were performed by two experienced endoscopists (PJJ, JMK) and surgeons (MYJ, JYJ), respectively. Written, informed consent was obtained from each patient included in the study. Gross type of EGCs were reviewed based on endoscopic images and defined by Paris classification [14]. ESD was performed if the lesion was estimated as within Gotoda’s expanded criteria; 1) differentiated type mucosal cancer, regardless of tumor size, 2) differentiated type with ulceration, tumor size ≤ 3.0 cm, 3) differentiated type with SM invasion ≤ 500 μm, tumor size ≤ 3.0 cm and 4) undifferentiated type mucosal cancer, tumor size ≤ 2.0 cm [15]. The presence of ulcer, tumor size, depth of invasion and lymphovascular invasion were determined by pathological evaluation of the resected specimen, which was fixed in 10% formalin and sectioned into 4-mm thick segments. Tumor differentiation was classified as differentiated or undifferentiated type according to the Japanese Gastric Cancer Treatment Guidelines (Version 4) [16]. The status of *H*. *pylori* infection was identified by special Wright-Giemsa stain. R0 resection was defined as a microscopically margin-negative resection, without gross or microscopic tumor remaining in the primary tumor bed [17]. Recurrence was defined as synchronous or metachronous cancer in the stomach detected by follow-up surveillance endoscopy.

### Statistical analysis

We performed statistical analysis using SPSS 20.0 (SPSS Inc., Chicago, IL, USA). Comparison of clinicopathological parameters between proximal and distal EGC was performed by Chi-squared test or Fisher’s exact test for categorical data and Student’s *t* test for continuous data. Multivariate analysis was performed using a stepwise multiple logistic regression model including statistically significant variables in univariate analysis. A *P* value less than 0.05 was considered as statistically significant.

## Results

### Baseline characteristics and clinicohistopathological outcomes

During the study period, a total of 1146 patients underwent endoscopic resection or surgery and diagnosed as EGC (endoscopy; 689, surgery 457). Among them, 600 cases were located on distal stomach (endoscopy; 414, surgery 186) and 119 on proximal stomach (endoscopy; 63, surgery; 56). We matched age and sex as 1:3 ratio, and finally enrolled 368 EGC patients including 92 patients assigned to the proximal group and 276 patients to the distal group. Baseline characteristics of the entire study population are summarized in Table 1. Age and sex were identical in both groups, and patients with a family history of gastric cancer, comorbidities except hypertension (, diabetes, cardiovascular disease, cerebrovascular disease, liver cirrhosis and chronic kidney disease), body mass index (BMI), past history of gastric cancer and *H*. *pylori* infection were not significantly different between the two groups. Procedure time was significantly prolonged in proximal group than distal group among the cases resected by ESD (58.2 ± 29.0 vs. 40.0 ± 31.7, *P* = 0.001), but not significantly different between both groups in patients with surgical resection. Among patients who underwent ESD, major bleeding in proximal and distal group was 5% (2/40) vs. 15% (33/220), and perforation was 5% (2/40) vs. 2.7% (6/220), which was not significantly different (*P* = 0.106). Among patients with surgical resection, major bleeding occurred only in proximal group (3.8%, 2/52), but not in distal group, despite no statistical significance (*P* = 0.139).

**Table 1:** Baseline characteristics of overall patients.

Variables	Proximal (n = 92)	Distal (n = 276)	Total (n = 368)	*P*-value
Age (year ± SD)	62.7 ± 11.1	62.6 ± 9.2	62.6 ± 9.7	0.939
Sex (male), n (%)	68 (73.9)	204 (73.9)	272 (73.9)	1.000
Family history of gastric cancer, n (%)	2 (2.2)	9 (3.3)	11 (3.0)	0.596
Comorbidity, n (%)				
Hypertension	41 (44.6)	86 (31.2)	127 (34.5)	0.019
Diabetes	18 (19.6)	52 (19.6)	70 (19.0)	0.878
Cardiovascular disease	6 (6.5)	15 (5.4)	21 (5.7)	0.697
Cerebrovascular disease	4 (4.3)	9 (3.3)	13 (3.5)	0.625
Liver cirrhosis	1 (1.1)	6 (2.2)	7 (1.9)	0.509
Chronic kidney disease	2 (2.2)	2 (0.7)	4 (1.1)	0.246
Past history of gastric cancer, n (%)	4 (4.3)	8 (2.9)	12 (3.3)	0.498
*Helicobacter pylori* infection, n (%)*	32 (58.2)	136 (60.7)	168 (60.2)	0.731

*Evaluation of *Helicobacter pylori* was performed 55 patients in proximal group and 224 patients in distal group

SD, standard deviation

In terms of histopathological outcomes, however, proximal EGCs were performed more frequently by surgery (52/92; 56.5% vs. 56/276; 20.3%, *P*<0.001), comprised more flat or depressed type (62/92; 67.4% vs. 136/276; 49.3%, *P* = 0.008), undifferentiated (35/92; 38.0% vs. 54/276; 19.6%, *P*<0.001), and diffuse types (23/92; 25.0% vs. 43/276; 15.6%, *P* = 0.041). The mean tumor size was 29.5 ± 19.4 mm in the proximal group, which was significantly larger than 20.3 ± 16.8 mm in the distal group (*P*<0.001), and EGCs larger than 30 mm were more frequently noted in the proximal group than in the distal group (41/92; 44.6% vs. 48/276; 17.4%, *P*<0.001). Furthermore, SM invasion was more frequently detected in the proximal group than in the distal group (56/92; 60.9% vs. 71/276; 25.7%, *P*<0.001). There was no significant difference in lymphovascular invasion between both groups. Among 260 patients who underwent endoscopic procedure, absolute, expanded and beyond expanded criteria judged by histopathologic evaluation were 30% (12/40), 35% (14/40) and 35% (14/40), respectively, in proximal group, and 63.2% (139/220), 20.9% (46/220) and 15.9% (35/220), respectively, in distal group. The distribution of ESD criteria in both groups were significantly different (*P*<0.001). 63 patients (24.2%) showed non-curative resection due to positive margin, presence of lymphovascular invasion or beyond expanded criteria of ESD by histopathologic evaluation. Among them, 7 patients had additional surgery, 3 had re-do ESD and 53 did not undergo additional procedure and were endoscopically followed-up. During the mean follow-up of 39.8 ± 24.1 months, the recurrence rate among patients who underwent ESD was 2.5% (1/40) in proximal group and 9.5% (21/220) in distal group. There was no recurrent case among patients who underwent surgical resection. After exclusion of 5 cases of synchronous cancers in distal group, two cases were local recurrence at ESD site and 15 cases were metachtonous cancers, thus recurrence rate was modified as 2.5% vs. 7.3%, which was not significantly different (*P* = 0.261). (Table 2). When we stratified the recurrence rate in cases with curative resection, neither was significantly different between proximal and distal group (1/20, 5.0% vs. 14/173, 8.1%, *P* = 0.625). In terms of nodal stage among patients who underwent surgery, N0, N1, N2 and N3 were 92.3% (48/52), 7.7% (4/52), 0% (0/52) and 0% (0/52), respectively, in proximal group, and 87.5% (49/56), 8.9% (5/56), 1.8% (1/56) and 1.8% (1/56), respectively, in distal group. The distribution of N stage in both groups were not significantly different (*P* = 0.577).

**Table 2:** Clinicopathologic outcomes between proximal and distal group.

Variables	Proximal (n = 92)	Distal (n = 276)	Total (n = 368)	*P*-value
Procedure type (ESD:surgery), n (%)	40:52 (43.5:56.5)	220:56 (79.7:20.3)	260:108 (70.7:29.3)	<0.001
Gross type, n (%)				0.008
Elevated	30 (32.6)	140 (50.7)	170 (46.2)	
Flat or depressed	62 (67.4)	136 (49.3)	198 (53.8)	
Ulcer, n (%)	9 (9.8)	50 (18.1)	59 (16.0)	0.059
En bloc resection, n (%)	91 (98.9)	275 (99.6)	366 (99.5)	0.189
R0 resection, n (%)	85 (92.4)	255 (92.4)	340 (92.4)	1.000
Histopathologic differentiation, n (%)				<0.001
Differentiated type	57 (62.0)	222 (80.4)	279 (75.8)	
Undifferentiated type	35 (38.0)	54 (19.6)	89 (24.2)	
Lauren classification, n (%)				0.041
Intestinal	69 (75.0)	233 (84.4)	302 (82.1)	
Diffuse	23 (25.0)	43 (15.6)	66 (17.9)	
Size (mm ± SD)	29.5 ± 19.4	20.3 ± 16.8	22.6 ± 17.9	<0.001
Size subcategory				<0.001
<20	32 (34.8)	172 (62.3)	204 (55.4)	
20≤ <30	19 (20.7)	56 (20.3)	75 (20.4)	
≥30	41 (44.6)	48 (17.4)	89 (24.2)	
Depth of invasion, n (%)				<0.001
Mucosa	36 (39.1)	205 (74.3)	241 (65.5)	
Submucosa	56 (60.9)	71 (25.7)	127 (34.5)	
Depth of mucosa cancer, n (%)				0.542
Lamina propria	22 (61.1)	136 (66.3)	158 (65.6)	
Muscularis mucosa	14 (38.9)	69 (33.7)	83 (34.4)	
Depth of submucosa cancer, n (%)*				0.279
SM1	20 (39.2)	21 (30.4)	41 (34.2)	
SM2	8 (15.7)	19 (27.5)	27 (22.5)	
SM3	23 (45.1)	29 (42.0)	52 (43.3)	
Lymphovascular invasion, n (%)	3 (3.3)	13 (4.7)	16 (4.4)	0.567
Follow-up duration (month ± SD)	39.1 ± 22.7	40.0 ± 24.7	39.8 ± 24.1	0.766
Recurrence, n (%)**	1 (2.5)	16 (7.3)	17 (6.5)	0.261

*Subdivision of submucosal invasion was not performed in 5 patients among proximal group and 2 patients in distal group

**Recurrence among patients who underwent ESD. Synchronous cancers were excluded.

SD, standard deviation, ESD, endoscopic submucosal dissection

### Risk factors for SM invasion

We evaluated the significant risk factors of SM invasion among the entire study population. Univariate analysis showed that proximal location, flat or depressed appearance, undifferentiated type, tumor size, diffuse type and lymphovascular invasion were significant risk factors for SM invasion. Multivariate analysis revealed that proximal location (odds ratio (OR), 3.541; 95% confidence interval (CI), 2.053–6.110; *P*<0.001), tumor size (20≤ <30: OR, 2.196; 95% CI, 1.190–4.054; *P* = 0.012); (≥30: OR, 3.388; 95% CI, 1.844–6.225; *P*<0.001) and lymphovascular invasion (OR, 4.885; 95% CI, 1.5216–15.745; *P*<0.001) were significant risk factors (Table 3).

**Table 3:** Risk Factors for submucosa invasive early gastric cancer among overall patients.

	Univariate analysis				Multivariate analysis			
Variables	*P*-value	Odds ratio	95% CI		*P*-value	Odds ratio	95% CI	
			Lower	Upper			Lower	Upper
Proximal location	<0.001	4.491	2.729	7.391	<0.001	3.541	2.053	6.110
Flat or Depressed	0.013	1.841	1.137	2.980	0.195	1.446	0.828	2.526
Ulcer	0.684	0.884	0.488	1.601	0.832	1.075	0.550	2.099
Undifferentiated type	<0.001	2.457	1.507	4.006	0.367	1.507	0.619	3.672
Size*								
20≤ <30	<0.001	2.870	1.624	5.074	0.012	2.196	1.190	4.054
≥30	<0.001	5.679	3.301	9.769	<0.001	3.388	1.844	6.225
Diffuse type	0.004	2.213	1.289	3.799	0.930	0.957	0.358	2.559
Lymphovascular invasion	0.006	4.515	1.533	13.299	<0.001	4.885	1.516	15.745

*Compared with size <20mm

CI, confidence interval

### Subgroup analysis of clinicohistopathological parameters: Size < 30mm

Tumor size varied significantly between the proximal and distal groups among the entire study population. To minimize the effect of tumor size on the invasiveness of EGC, we performed subgroup analysis by selecting EGCs less than 30 mm, including 51 patients from the proximal and 228 patients from the distal groups. Clinicohistopathological variables are listed in Table 4. Age, gender, BMI and tumor size were not significantly different in both groups. However, Procedure time was significantly prolonged among the cases resected by ESD (58.9 ± 28.2 vs. 37.1 ± 30.1, *P*<0.001), surgery was frequently performed (15/51; 29.4% vs. 28/228; 12.6%, *P* = 0.002), and flat or depressed type was significantly found (15/51; 29.4% vs. 28/228; 12.6%, *P* = 0.002) in the proximal group than in the distal group. In terms of histopathological parameters, undifferentiated type (16/51; 31.4% vs. 32/228; 14.0%, *P* = 0.003) and SM invasion (29/51; 56.9% vs. 45/228; 19.7%, *P*<0.001) were more frequent in the proximal group, which was consistent with the outcomes from the entire study population. Recurrence rate among patients who underwent ESD and tumor size < 30 mm were 2.8% (1/36) in proximal group and 8.0% (16/200) in distal group, which was not significantly different (*P* = 0.265).

**Table 4:** Clinicopathologic characteristics and outcomes in subgroup: Tumor size < 30 mm.

Variables	Proximal (n = 51)	Distal (n = 228)	Total (n = 279)	*P*-value
Sex (male), n (%)	41 (80.4)	173 (75.9)	214 (76.7)	0.490
Age (year ± SD)	63.1 ± 10.1	62.4 ± 9.0	62.8 ± 9.5	0.642
*Helicobacter pylori* infection, n (%)*	20 (50.0)	119 (63.6)	139 (61.2)	0.108
Procedure type (ESD:surgery), n (%)	36:15 (70.9:29.4)	200:28 (87.7:12.3)	236:43 (84.6:15.4)	0.002
Gross type, n (%)				0.035
Elevated	19 (37.3)	121 (53.1)	140 (50.2)	
Flat or depressed	32 (62.7)	107 (46.9)	139 (49.8)	
Ulcer, n (%)	6 (11.8)	42 (18.4)	48 (17.2)	0.255
En bloc resection, n (%)	51 (100.0)	227 (99.6)	278 (99.6)	0.636
R0 resection, n (%)	44 (86.3)	215 (94.3)	259 (92.8)	0.045
Histopathologic differentiation, n (%)				0.003
Differentiated type	35 (68.6)	196 (86.0)	231 (82.8)	
Undifferentiated type	16 (31.4)	32 (14.0)	48 (17.2)	
Lauren classification, n (%)				0.224
Intestinal	42 (82.4)	202 (88.6)	244 (87.5)	
Diffuse	9 (17.6)	22 (11.4)	35 (12.5)	
Size (mm ± SD)	15.9 ± 6.4	14.3 ± 6.9	14.9 ± 6.7	0.100
Depth of invasion, n (%)				<0.001
Mucosa	22 (43.1)	183 (80.3)	205 (73.5)	
Submucosa	29 (56.9)	45 (19.7)	74 (26.5)	
Lymphovascular invasion, n (%)	0 (0.0)	10 (4.4)	10 (3.6)	0.128
Recurrence, n (%)	1 (2.8)	16 (8.0)	17 (7.2)	0.265

*Evaluation of *Helicobacter pylori* was performed 40 patients in proximal group and 187 patients in distal group

SD, standard deviation, ESD, endoscopic submucosal dissection

### Risk factors of SM invasion among subgroups: Size < 30mm

We also performed univariate and multivariate analyses to detect the risk factors for SM invasion among subgroups with size < 30 mm using a multiple logistic regression analysis model. Proximal tumor location (OR, 5.940; 95% CI, 2.974–11.862; *P*<0.001), flat or depressed appearance (OR, 2.184; 95% CI, 1.089–4.379; *P* = 0.028) and lymphovascular invasion (OR, 8.487; 95% CI, 2.224–32.979; *P* = 0.002) were significant risk factors for SM invasion among subgroups (Table 5).

**Table 5:** Risk factors for submucosa invasive early gastric cancer in subgroup: Tumor Size < 30 mm.

	Univariate analysis				Multivariate analysis			
Variables	*P*-value	Odds ratio	95% CI		*P*-value	Odds ratio	95% CI	
			Lower	Upper			Lower	Upper
Proximal location	<0.001	5.361	2.818	10.197	<0.001	5.940	2.974	11.862
Flat or depressed	0.037	1.921	1.041	3.544	0.028	2.184	1.089	4.379
Ulcer	0.793	0.909	0.444	1.858	0.720	0.862	0.383	1.938
Undifferentiated type	0.001	2.913	1.526	5.559	0.279	1.930	0.588	6.337
Diffuse type	0.007	2.701	1.305	5.589	0.706	1.295	0.337	4.976
Lymphovascular invasion	0.024	4.434	1.215	16.183	0.002	8.487	2.224	32.979

CI, confidence interval

## Discussion

In this study, we demonstrated that proximal EGCs were significantly larger, showed undifferentiated and diffuse type, and invaded SM more frequently compared with distal EGCs in an age and sex-matched cohort. Furthermore, the proximal location of EGC was a significant risk factor of SM invasion in the overall population as well as subgroups with tumor size < 30 mm. Based on our results, we recommend careful pre-procedural evaluation and consideration of optimal procedure in case of proximal EGCs. Previous studies investigated different clinicopathological characteristics of proximal gastric cancers by comparing distal or non-proximal gastric cancers, however, most of the cases included in these studies were advanced gastric cancers (AGCs) corresponding to T2~4 stage [18–22], and many confounding variables were not well adjusted. The strength of our study relates to inclusion of EGCs confined to mucosa or submucosa layer, in other words, T1 cancer. Furthermore, we reduced confounding risk factors such as age and male sex by pre-analysis matching. Many Western and Eastern studies demonstrated that proximal gastric cancer is significantly frequent in advanced age and male patients compared with distal gastric cancer [23–29]. Thus, we matched proximal EGC cases with distal EGCs by age and sex to minimize the confounding effects of baseline characteristics. After adjustment, however, several significant histopathological features were still observed in patients with proximal EGCs, which suggest the unique biological behavior of proximal EGCs. SM invasion is a critical concern for endoscopic treatment of EGCs, because SM invasion is one of the most common causes of failure of curative resection of ESD (defined by both *en bloc* and complete resection without lymphovascular invasion and meeting the expanded criteria of ESD), which significantly leads to tumor recurrence during long-term follow-up [30]. Pre-ESD evaluation may provide predictive information for SM invasion of EGC, including endoscopic (subepithelial tumor-like marginal elevation, fusion of convergent folds, irregular nodularity or submucosal fibrosis) [31] or EUS findings (blurring, obliteration or infiltration or SM layer) [32]. However, diagnostic accuracy of these procedures is highly dependent on the endoscopist, and inter-observer variation is an important challenge. An image enhanced endoscopy such as magnifying endoscopy with narrow-band imaging (ME-NBI) is attracting attention as a new alternative diagnostic tool that predict SM invasion of EGCs. A recent study showed that the presence of dilated vessel detected by ME-NBI predicted SM invasion with 81.5% of diagnostic accuracy and 88.3% of specificity [33]. The reason for predominant SM invasion of proximal EGC is rarely known. The thickness of stomach wall varies according to the location, and is thicker in the antrum compared with body and cardia. Subsequently, the SM layer is thinner in the proximal location than in the distal location [34]. Furthermore, thickness of mucosal layer is often thicker in antrum than proximal body or cardia [35], which may contribute to the predominance of mucosal cancer in distal group than proximal group. A recent Chinese data showed that gastric carcinoma with lymphoid stroma is significantly found in proximal than distal EGCs, which may link to greater tendency toward SM invasion [36]. Other molecular mechanisms may be involved in the invasiveness of proximal EGCs, which need to be further investigated.

Interestingly, undifferentiated and diffuse type EGCs were more frequently detected in the proximal group than in the distal group, which shows discrepancy with data from previous studies. Studies that compared proximal and distal gastric cancers generally showed that differentiated and intestinal type were predominant in proximal gastric cancer compared with non-proximal gastric cancer [19, 21, 22]. However, predominant type of tumor differentiation might vary across different regions and countries. Several studies included AGC patients as well as EGC that differed from our study population, which may affect the discrepant results from our study in terms of tumor differentiation and Lauren’s classification. A large-scale retrospective Korean study demonstrated that undifferentiated and diffuse type were significantly frequent in the upper third gastric cancer than the middle or distal gastric cancer, which reinforces our study results [37]. We considered that predominance of undifferentiated type, as well as frequent SM invasion, in proximal stomach might be another characteristics of biologic behavior of proximal EGC, because undifferentiated EGCs usually show aggressive histologic findings with deeper invasion depth even in case with relatively small tumor size [38].

The tumor size was also an important parameter with significant differences between both groups in our study, which is consistent with previous data. Endoscopically, the size of the tumor is often underestimated in the proximal EGC compared with the distal EGC, and tumor margin is often ambiguous in the case of undifferentiated EGC [39]. The biological behavior of proximal EGC, and the predominance of undifferentiated and diffuse types in proximal EGC of our study population may lead to significant differences in tumor size between both groups. To minimize the influence of tumor size, we performed subgroup analysis by sorting EGCs measuring < 30 mm. The proximal EGCs also showed undifferentiated type and SM invasion more frequently, and proximal location remained a significant risk factor for SM invasion among the subgroups.

Our study has several limitations. First, due to fundamental limitation of observational study, other important confounding variables such as dietary factors and smoking were not adjusted although we matched age and gender before analysis. Second, the status of *H*. *pylori* infection, the most crucial risk factor of gastric cancer, was not investigated in entire study population, and the success or failure of eradication among infected patients was not analyzed. Third, therapeutic procedure of EGC was not uniformly performed. The patient with ESD and those with surgery were mixed and surgery was more frequently performed in the proximal group, which may have affected the difference in recurrence rate according to the location of EGC. Forth, among 63 patients with non-curative resection after ESD, 53 patients did not undergo additional resection due to old age, comorbidities or patients’ refusal, which may weaken accurate assessment of histopathology and prognosis.

In conclusion, our study data suggest that proximal EGCs may exhibit different clinicopathological characteristics and more aggressive biological behavior such as larger size and SM invasion compared with distal EGCs. Further investigation of characteristics of proximal EGC including genetic and molecular signature is needed in the future.

## Supporting information

S1 Table Raw data of study. (Proximal_raw data_plosone.).
(XLSX)

## Abbreviation

ESD: endoscopic submucosal dissection
EGC: early gastric cancer
SM: submucosal
EUS: endoscopic ultrasonography
WLE: white-light endoscopy
EGJ: esophagogastric junction
EAC: esophageal adenocarcinoma
*H*. *pylori*: *Helicobacter pylori*
BMI: body mass index
OR: odds ratio
CI: confidence interval
AGC: advanced gastric cancer
ME-NBI: Magnifying endoscopy with narrow-band imaging
